# Anastomotic biliary stricture after orthotopic liver transplantation: what can we propose after failure of classic ERCP technique to cannulate the stricture?

**DOI:** 10.1055/a-2248-6398

**Published:** 2024-02-15

**Authors:** Pierre Mayer, Pierre-Yves Christmann, Lucile Héroin, François Habersetzer

**Affiliations:** 1 Department of Gastroenterology and Hepatology, Pôle Hépato-digestif, Nouvel Hôpital Civil, Les Hôpitaux Universitaires de Strasbourg (HUS), Strasbourg, France; 2 Digestive Endoscopy Unit, IHU-Strasbourg (Institut Hospitalo-Universitaire), Strasbourg, France; 3 Inserm U1110, Institute for Viral and Liver Diseases, LabEx HepSYS, University of Strasbourg, Faculty of Medicine, Strasbourg, France

Liver transplantation is the only curative treatment for severe liver disease. Unfortunately, there are several complications associated with liver transplantation. Biliary complications occur in around 15%–30% of cases, with an estimated mortality rate of 10% 1 2 3 .

We report the case of a 66-year-old patient, with a history of recent liver transplantation for alcoholic cirrhosis. He presented with the progressive onset of jaundice associated with disturbances in hepatic tests. Magnetic resonance imaging revealed a biliary anastomosis stricture, with an upstream dilated common bile duct (17 mm) ( Fig. 1 , Fig. 2 ). We therefore performed an endoscopic retrograde cholangiopancreatography (ERCP).

**Fig. 1:**
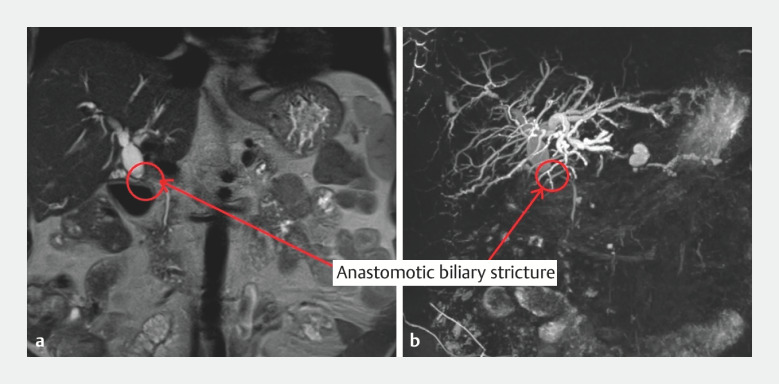
Coronal section magnetic resonance imaging (MRI) image showing a disparity in caliber between the recipient bile duct and the graft bile ducts. **a** T2 MRI sequence showing anastomotic stricture with dilatation of the upstream bile ducts (circle and red arrow). **b** Three-dimensional MRI reconstruction showing anastomotic stricture with dilatation of upstream bile ducts (circle and red arrow).

**Fig. 2:**
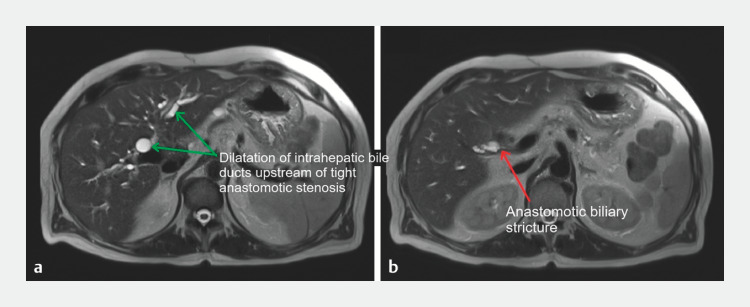
Cross-sectional magnetic resonance imaging (MRI) image showing a disparity in caliber between the recipient bile duct and the graft bile ducts. **a** T2 MRI scan showing dilatation of the common hepatic duct and intrahepatic bile ducts (green arrows). **b** T2 MRI scan showing biliary anastomotic stricture (red arrow).

Biliary cannulation and endoscopic sphincterotomy presented no difficulty. Biliary opacification revealed a short, very tight anastomotic stricture ( Fig. 3 ). However, despite several attempts, it was impossible under fluoroscopy guidance to pass a 0.035-inch or 0.025-inch angled and straight guidewire through the stricture. Given the impossibility of positioning a guidewire through the stricture with the standard technique, we decided to perform a single operator cholangioscopy (SOC).

**Fig. 3:**
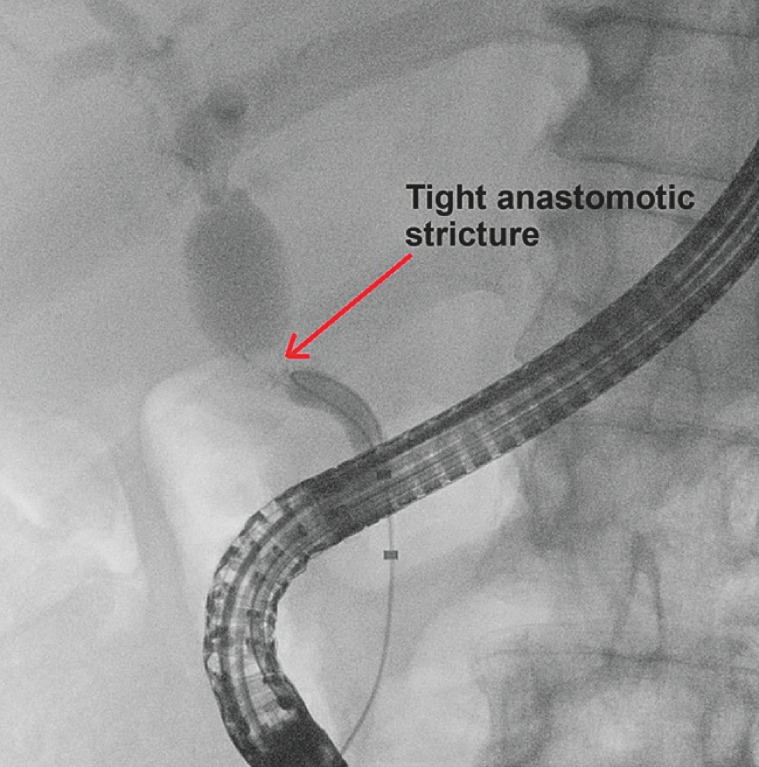
Retrograde cholangiography showing tight stricture of the biliary anastomosis (red arrow).

The use of SOC revealed a punctiform benign stricture with an anastomotic recess ( Fig. 4 ). A 0.025-inch straight guidewire was then used, and could be inserted into the right intrahepatic bile ducts under endoscopic and fluoroscopic guidance ( Video 1 ). The stricture was then dilated with a 6-mm balloon, followed by placement of a 10×80 mm covered metal stent ( Fig. 5 ).

**Fig. 4:**
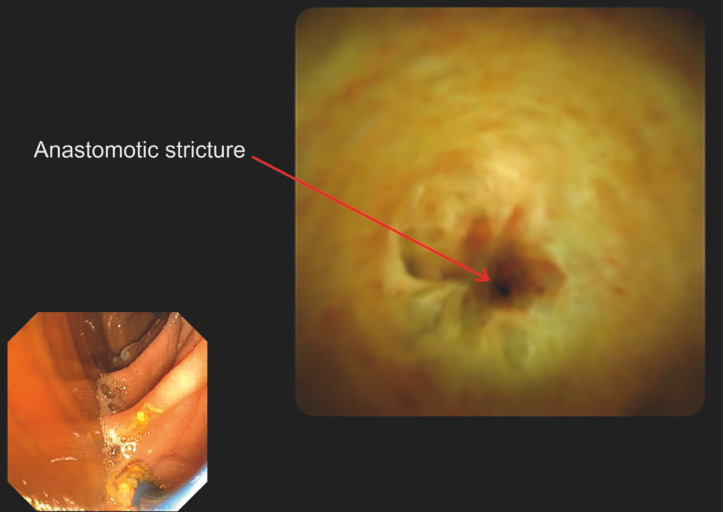
Cholangioscopy image showing punctiform stricture at the biliary anastomosis, with perianastomotic recess.

Use of single operator cholangioscopy for selective cannulation of punctiform anastomotic stricture post liver transplantation, after failure of cannulation with standard endoscopic retrograde cholangiopancreatography techniques.Video 1

**Fig. 5:**
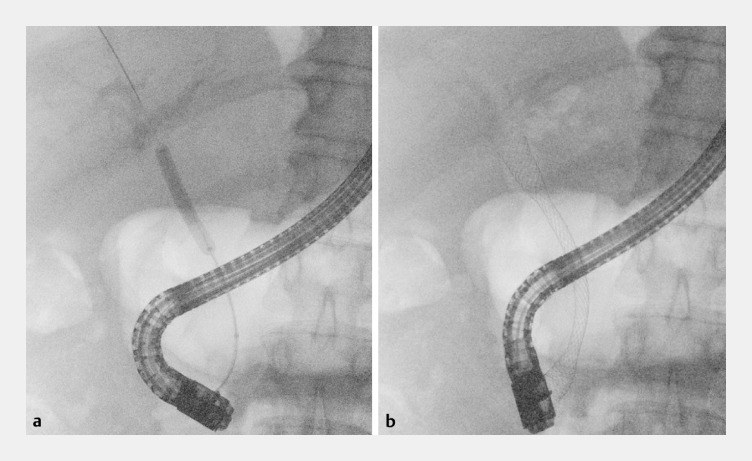
Endoscopic retrograde cholangiopancreatography after cannulation of the stricture under cholangioscopy. **a** Balloon dilatation of the anastomosis. **b** Placement of a 10 × 80 mm covered metal stent.

Few studies in the literature focus on the contribution of SOC as a therapeutic device (such as selective cannulation of the bile ducts under endoscopic control) apart from lithotripsy. SOC probably has its place in the therapeutic management of complex biliary strictures, enabling selective cannulation of the area to be drained or to cross complex strictures such as those occurring after post liver transplantation, as illustrated in this case 4 5 .

Endoscopy_UCTN_Code_TTT_1AR_2AG

## Notes

Endoscopy E-Videos https://eref.thieme.de/e-videos
*E-Videos* is an open access online section of the journal *Endoscopy* , reporting on interesting cases and new techniques in gastroenterological endoscopy. All papers include a high-quality video and are published with a Creative Commons CC-BY license. Endoscopy E-Videos qualify for HINARI discounts and waivers and eligibility is automatically checked during the submission process. We grant 100% waivers to articles whose corresponding authors are based in Group A countries and 50% waivers to those who are based in Group B countries as classified by Research4Life (see: https://www.research4life.org/access/eligibility/ ). This section has its own submission website at https://mc.manuscriptcentral.com/e-videos .
